# Revisiting the follicle-stimulation hormone receptor expression and function in human myometrium and adipose tissue

**DOI:** 10.1186/s10020-024-01015-2

**Published:** 2024-12-04

**Authors:** Ewelina Palak, Donata Ponikwicka-Tyszko, Kamila Pulawska-Moon, Maria Sztachelska, Gabriela Milewska, Beata Modzelewska, Tomasz Kleszczewski, Maria L. Koivukoski, Piotr Bernaczyk, Hady Razak Hady, Piotr Gołaszewski, Aleksandra N. Lupinska, Marek Kulikowski, Adam Lemancewicz, Ilpo T. Huhtaniemi, Slawomir Wolczynski, Nafis A. Rahman

**Affiliations:** 1 Department of Biology and Pathology of Human Reproduction, Institute of Animal Reproduction and Food Research, Polish Academy of Sciences, Olsztyn, 10-748 Poland; 2 Institute of Biomedicine, Research Centre for Integrative Physiology and Pharmacology, University of Turku, Turku, 20520 Finland; 3 Department of Reproduction and Gynecological Endocrinology, Medical University of Bialystok, Bialystok, 15-276 Poland; 4 Department of Biophysics, Medical University of Bialystok, Bialystok, 15-269 Poland; 5 Department of Medical Pathomorphology, Medical University of Bialystok, Bialystok, 15-269 Poland; 6 1st Clinical Department of General and Endocrine Surgery, Medical University of Bialystok, Bialystok, 15- 269 Poland; 7 Department of Perinatology, Medical University of Bialystok, Bialystok, 15-269 Poland; 8 Institute of Reproductive and Developmental Biology, Imperial College London, London, W12 0NN UK

**Keywords:** FSHR, Myometrium, Adipose tissue, Adipocytes, Contractile activity

## Abstract

**Background:**

Extragonadal follicle-stimulating hormone receptor (FSHR) expression at low levels has been shown in several normal and tumor tissues, including myometrium and adipose tissue. FSH-FSHR signaling in the myometrium has been suggested to regulate uterine contractile activity and the timing of labor. In contrast, FSH-FSHR has been linked to the activation of brown/beige fat thermogenesis in adipose tissue. The issue of extragonadal FSHR expression and its functionality remains contentious within the scientific community, as contradictory findings necessitate further independent and critical analyses. Hereby, we re-investigated the FSHR expression and its functionality in normal non-pregnant (M-NP) and pregnant (N-P) human myometrium, as well as in human visceral (VAT) and subcutaneous (SCAT) adipose tissue (AT).

**Methods:**

FSHR expression at mRNA (real-time qPCR, RNAscope in situ hybridization) and protein (immunohistochemical staining) levels in adipose tissue, myometrium, and adipocytes were evaluated. Myometrium and adipocytes were treated with recombinant (rh)FSH to study its effects on functional pathways. Myometrium contractile activity was measured using a force transducer with digital output and the DASYLab software unit. Cyclic adenosine monophosphate (cAMP) production by myometrium explants and adipocytes was measured using a cAMP ELISA Kit. The activation of the AKT pathway in myometrium and adipocytes was analyzed by Western blot analysis.

**Results:**

Contrary to previous observations, we found no expression of FSHR at either mRNA or protein levels in M-NP, N-P, VAT, and SCAT. Treatment with recombinant human FSH (rhFSH) showed no effect on cAMP production or phosphorylation of AKT in M-NP, N-P, as well as in VAT and SCAT. rhFSH treatment did not influence contractile activity in M-NP, N-P.

**Conclusions:**

These findings suggest that the FSHR signaling pathway does not regulate myometrial contractility during pregnancy. Additionally, the absence of FSHR expression in both VAT and SCAT implied that FSHR does not play a role in the functional signaling pathways in adipose tissues. In conclusion, our findings contradict earlier data on the involvement of FSH-FSHR signaling in regulating myometrial contractility near term, as well as in adipose tissue function.

**Supplementary Information:**

The online version contains supplementary material available at 10.1186/s10020-024-01015-2.

## Introduction

Follicle-stimulating hormone (FSH) is a heterodimeric glycoprotein hormone produced by the anterior pituitary gland (Griswold et al. 1995). The transduction of the FSH-induced signal is mediated by the FSH-specific G protein-coupled receptor (FSHR), expressed mainly in ovarian granulosa and testicular Sertoli cells (Griswold et al. 1995). In the canonical pathway, FSH binds to the extracellular domain of FSHR and activates the G_αs_/cAMP/PKA signaling pathway (Means et al. 1974; Dattatreyamurty et al. 1987). FSH may also act through the PI3K/PIP3–AKT/mTOR pathway, β-arrestin-dependent pathway, or interact FSHR with PPL1, FoxO1a and 14-3-3τ (Gloaguen et al. 2011). FSH-FSHR signaling plays a key role in sexual development and reproduction in both males and females (Matthews et al. 1993; Lindstedt et al. 1998).

Several studies have demonstrated the expression of FSHR in extragonadal tissues (Kim et al. 2024) including normal secretory-phase endometrium and endometriotic lesions (Ponikwicka-Tyszko et al. 2016), myometrium (Stilley et al. 2014a, 2016), placenta, fallopian tube (Stilley et al. 2014a), cervix (Stilley et al. 2014a), human umbilical cord endothelial cells (Stilley et al., 2014b), bone osteoclasts (Sun et al. 2006; Zhu et al. 2012), adipose tissue (Liu et al. 2015, 2017), as well as in tumor endothelial cells of different human cancers and their metastases (Siraj et al. 2013). FSHR expression has also been demonstrated in muscle fibers and stromal cells of human pregnant term myometrium (M-P) (Stilley et al. 2014a, 2016). It has been suggested that higher myometrial FSHR levels dictate the quieting versus stimulation of myometrial contractile activity near the term of pregnancy in response to FSH, but the exact mechanism remains unknown (Stilley et al. 2014a).

Adipose tissue (AT) plays a central role in energy homeostasis by acting as a major lipid storage site as well as an important endocrine organ (Kershaw and Flier 2004). The two main types of adipose tissue are white (WAT) and brown adipose tissue (BAT). WAT stores excess energy as triglycerides (TGs), and produces hormones and cytokines that regulate body metabolism, while BAT generates body heat via thermogenesis (Cypess et al. 2009; Saito et al. 2009; Marken Lichtenbelt et al. 2009; Nedergaard et al. 2007; Richard et al. 2020). WAT is divided into visceral fat (VAT), which is located around internal organs, and subcutaneous fat (SCAT), located beneath the skin (Lee et al. 2013). Individual VAT/SCAT depots differ in structure, function, gene expression profile, and metabolic and endocrine activity. Several cohort studies on perimenopausal and postmenopausal women have shown a positive correlation between serum FSH levels, waist-to-hip ratio, and fat mass (Sowers et al. 2007; Gavaler and Rosenblum 2003; Seth et al. 2013). In human and mouse adipose tissues, FSHR expression at mRNA and protein levels has been reported in VAT, SCAT, and adipocytes (Liu et al. 2015, 2017). Blockage of the FSH signaling with a specific polyclonal FSH antibody has been shown to reduce total body fat by activating brown/beige fat thermogenesis (Liu et al. 2017; Gera et al. 2020). However, other FSH/LH blockage studies have failed to show any body fat loss in mice and humans treated with GnRH agonists/antagonists (Vuorenoja et al. 2009; Doroszko et al. 2019).

Recent extragonadal FSHR expression findings opened up novel possibilities for functional FSHR as a diagnostic, prognostic, and therapeutic tool for cancer therapy (Siraj et al. 2013; Radu et al. 2010; Sheng et al. 2022; Starzynski et al. 2023), potentially also for postmenopausal obesity (Liu et al. 2015, 2017) bone mass/osteoporosis (Sun et al. 2006; Zhu et al. 2012), angiogenesis (Stilley et al., 2014b) or Alzheimer disease (Xiong et al. 2022). FSHR signaling pathway was suggested to regulate myometrial contractility and labor progression (Stilley et al. 2016), and regulating obesity (Liu et al. 2015, 2017). However, only reliable and independent confirmation of the FSHR presence may provide a basis for further research explaining the underlying mechanisms and potential applications. The revision of FSHR expression in some extragonadal tissue has revealed earlier no FSHR transcripts or gene amplification (Stelmaszewska et al. 2016; Chrusciel et al. 2019; Tedjawirja et al. 2023a, b). Due to these discrepancies, the extragonadal FSHR expression should be critically re-examined using additional methods subjected to proper validation (Ponikwicka-Tyszko et al. 2016; Stelmaszewska et al. 2016; Chrusciel et al. 2019; Tedjawirja et al. 2023a, b). We revisited the FSHR expression and function in uterine and adipose tissue, along with an array of positive and negative controls. Because of the existing uncertainty surrounding the studies on extragonadal FSHR expression, it is important that also alternative/additional independent and critical findings on this controversial topic be reported.

## Materials and methods

### Patients and collection of tissue samples

Adipose tissues (N = 25 VAT, N = 25 SCAT, N = 25 polycystic ovary syndrome (PCOS) VAT) were obtained from patients undergoing surgery at the Medical University of Bialystok. Visceral and subcutaneous adipose tissues were collected from obese (BMI > 30) and lean (BMI < 25) non-PCOS women (patient age range, 25–65 years). PCOS VAT was collected from lean age and BMI matched with control group patients. This study has been approved by the local Human Investigation Ethics Committee at the Medical University of Bialystok (opinion no. R-I-002/147/2018). Written informed patient’ consent has been obtained from each patient.

Biopsies of human uterine muscle tissue during pregnancy were obtained during emergency cesarean section operations at the Medical University of Bialystok which was done due to other reasons than the absence of spontaneous contractile activity. The Bioethics Committee of the Medical University of Bialystok previously approved the study protocol (Opinion No.R-I-002/283/2019). Written informed patient consent was obtained from each patient. Myometrium was collected from 15 pregnant women aged 20–36 years. The BMI of pregnant patients was < 25, with no history of PCOS, and no gestational diabetes. Normal control group myometrium (*n* = 25) was obtained from women aged 40–45 during hysterectomy which was done due to other reasons than uterine myoma. All control myometrium and adipose tissue samples were collected during surgical procedures performed in the follicular phase. All the women included in the study were Caucasian and came from cities. Patients with any malignant, hormonally active processes in the genital tract, hormonal treatment during the last 6 months, menopause, and hormonal disorders were excluded from the study. The study was conducted per the principles of the Helsinki Declaration of the World Medical Society, the International Conference on Good Clinical Practice Harmonization Guidelines, and Polish laws and regulations.

### Adipocyte isolation and differentiation

Adipocytes were isolated according to the previously published protocol (Ge et al. 2016). The samples were sliced into small pieces (diameter 0.5 cm), transferred into a 50 ml tube, and digested with 1 mg/ml Collagenase type I solution (Sigma-Aldrich, St. Louis, MO, USA, cat. no: C9891-1G) at 37 °C incubator with shaking for 1.5 h. Collagenase was inactivated by adding DMEM/F-12 medium with phenol red (GIBCO, Paisley, UK, cat. no. 11330032) supplemented with 20% fetal bovine serum (FBS; GIBCO, cat. no. A5256701), 100 units/ml penicillin and 100 µg/ml streptomycin (P/S solution; Sigma-Aldrich, cat no. P4333). To remove debris the cell suspension was filtered through a 100 μm nylon mesh cell strainer and centrifuged at 800 x g for 10 min at room temperature. The cell suspension was transferred onto a cell culture-treated flask and cultures were incubated at 37 °C in humidified atmosphere in the presence of 5% CO2 until they reached 70% confluence. The cells were seeded in a DMEM/F12 culture medium to 6-well plates (Sarstedt, Newton, NC) at 10 000 cells/cm^2^ density. Then cells were treated with DMEM/F-12 medium with phenol red supplemented with 10% FBS and P/S solution containing 1 µM dexamethasone (Sigma-Aldrich, cat. no. D4902), 58 µg/ml insulin (Sigma-Aldrich, cat. no. I0516), 0.5 mM IBMX (Sigma-Aldrich, cat. no. I5879) and 200 µM indomethacin (Sigma-Aldrich, cat. no. I7378) for 2 weeks. The medium was changed every 3 days. After this time cells were treated with a medium containing 10 µg/ml insulin. The medium was changed every 2 days. Cells were assayed after 6 days post-treatment with insulin medium. To confirm that adipocytes differentiate properly, we checked the expression of mature adipocytes markers (Ghaben and Scherer 2019): peroxisome proliferator-activated receptor gamma (PPARG), CCAAT enhancer binding protein alpha (CEBP/A), CCAAT enhancer binding protein beta (CEBP/B), adiponectin (ADIPOQ), leptin (LEP), lipoprotein lipase (LPL) and perilipin 1 (PLIN1).

### Myometrium tissue processing and data acquisition

The biopsies of outer myometrium were excised from the upper lip of the lower uterine segment, incision in the midline, i.e., the upper portion of the lower uterine segment. Immediately after collection, the samples were placed in ice-cold Tyrode’s solution and transferred to the laboratory, where they were processed as previously described (Modzelewska et al. 2003). Briefly, 4–8 strips, 0,6 − 0,7 cm in length, and a 2 × 2 mm area cross section were obtained under a dissecting microscope. The strips were then mounted in an isolated organ bath containing 20 ml of Tyrode’s solution (NaCl 136.9 mM; KCl 2.70 mM; MgCl2 1.05 mM; NaH2PO4 1.33 mM; CaCl2 1.80 mM; NaHCO3 25.0 mM; and glucose 5.0 mM) thermostatically maintained at 37ºC, pH 7.4, and saturated with carbogen (95% O2 + 5% CO2). The myometrium strips were left for an equilibration period of 1–2 h, in which the tensioning force was adjusted to 2 mN. After this time, regular phase contractions were achieved in tissues from 5 patients. In the case of tissues collected from the remaining 5 patients as the control group, there were no regular phase contractions, and oxytocin (OXT, oxytocic hormone, Sigma-Aldrich, cat. no. O3251) was administered to the bath at a concentration of 10^− 6^ mol/L.

The strength of the myometrium tension was recorded using a force transducer with digital output (BIO-SYS-TECH, Bialystok, Poland) and with the DASYLab software unit (version 9.0; Laboratory Data Acquisition System, SuperLogics, Waltham, MA, USA). Before each experiment, the strips were activated by 80 mmol/L K^+^. Appropriate controls were run under similar experimental conditions in strips of uterus obtained from the same woman.

The recombinant human (rh)FSH (R&D Systems, Minneapolis, MN, USA, cat. no. 5925-FS) was added cumulatively to the organ chambers in increasing concentrations of 1, 10, 100, and 1000 IU/L at 10-minute intervals, and the effects were recorded. Only one dose-response curve was made for each myometrial strip.

The myometrial responses to the substances administered were quantified based on the area under the curve (AUC). AUC was measured as the area under the curve showing the myometrial tensile force 10 min after adding test substances (i.e. OXT and FSH) (Cheung et al. 2005).

### Measurements of contraction parameters

The contractile activity of the myometrial strips before administering rhFSH (i.e., spontaneous or contractile activity after the administration of OXT at a concentration of 10^− 6^ mol/L) was treated as a control (set as 100%). The AUC was evaluated by calculating the integral of the appropriate curve section.

### cAMP production

Adipocytes were seeded onto 12-well plates (35000 cells/well) and grown overnight in a DMEM/F-12 medium with phenol red supplemented with 10% FBS. Myometrium explants from pregnant women were cut into 1-mm diameter pieces, plated onto 12-well plates, and maintained in a DMEM/F-12 medium with phenol red supplemented with 10% FBS overnight. Before the 1 h stimulation without (control) or with 10 (0.73 ng/mL), 100 (7.3 ng/mL), 1000 (73 ng/mL) IU/L rhFSH or 10 µM forskolin (FRK, Sigma-Aldrich, cat. no. F6886, used as a positive control), cells/explants were starved for 4 h in DMEM/F12 medium without phenol red (Gibco, cat. no. 21041025) with 0,5% hormone-depleted FBS (dFBS; Biochrom, Berlin, Germany) and P/S solution. According to the manufacturer’s instructions, FSH-stimulated cAMP production was evaluated using a cyclic AMP ELISA Kit (Cayman Chemical, MI, USA, cat. no. 581001).

### RNA isolation and gene expression

Total RNA was extracted from the cells using the TRIzol/chloroform method (Invitrogen, Carlsbad, CA, USA, cat. no. 15596018). The samples were mixed with chloroform (POCH, Gliwice, Poland, cat. no. 234431116) and centrifuged. Then total RNA was precipitated with 2-propanol (POCH, cat. no. 751500111) and washed thrice with 75% ethanol (POCH, cat. no. 396480111). The pellets were suspended in RNAse-free water (Invitrogen, Carlsbad, CA, USA, cat. no. AM9932). NanoDrop (Thermo Scientific, Massachusetts, USA) at 260 nm and gel electrophoresis determined the quality and quantity of isolated RNA. One µg of total RNA was incubated for 15 min with DNase I (Invitrogen, cat. no. 18068015) at room temperature, then the enzyme was deactivated by the addition of 1 µl of 25mM EDTA solution and incubation in 65 °C for 10 min. A commercially available SensiFAST™ cDNA Synthesis Kit (Meridian Bioscience Inc., Cincinnati, Ohio, USA, cat. no. BIO-65054) was used to carry out the reverse transcription reaction with the following reaction conditions: 25 °C for 10 min, 42 °C for 15 min, 48 °C for 15 min, 85 °C for 5 min. Gene expression analysis was performed on the StepOnePlus™ Real-Time PCR System (Applied Biosystems, Foster City, CA). The analysis of gene expression was performed using the housekeeping gene peptidylprolyl isomerase (PPIA) as a reference (Supplemental Table 1). For real-time qPCR, SYBR Green PCR master mix (Applied Biosystems, Foster City, CA, cat. no. 4309155) was used with the following reaction conditions: 2 min at 50 °C/ 10 min at 95 °C for initial denaturation and 15 s at 95 °C/ 1 min at 60 °C for a total of 40 amplification cycles. Every PCR reaction product was separated and verified by sequencing analysis.

### Immunohistochemical staining

Formalin-fixed paraffin-embedded tissue samples were deparaffinized and hydrated. Slides were boiled for 15 min in antigen retrieval buffer (10 mM citric acid buffer with 0.05% Tween20, pH = 6). Then, the sections were incubated in a humidified chamber for 1 h at RT with 3% BSA to reduce nonspecific background staining. Next, slides were incubated overnight in a humidified chamber at 4 ºC with the primary FSHR323 antibody (kindly provided by Dr. N. Ghinea; 1–5 µg/mL). Endogenous peroxidase activity was blocked by incubating slides in 0,5% H_2_O_2_ in TBS at room temperature for 20 min. DAKO polymer (DAKO EnVision + System – HRP labeled polymer; Agilent, Santa Clara, CA, cat. no. K4065) was applied onto each section and incubated in a humidified chamber for 30 min at room temperature. DAB + Chromogen (DAKO) was applied for 5 min. Slides were washed in dH2O, counterstained in Mayer’s hematoxylin (Sigma-Aldrich, cat. no. MHS1), dehydrated, and mounted with Pertex (Histolab Products, Göteborg, Sweden). As a control for the antibodies, tissues were incubated with 3% BSA and DAKO polymer to differentiate unspecific from specific staining. The IHC slides were digitally scanned by a P1000 Pannoramic scanner (3DHistech, Budapest, Hungary).

### RNAscope in situ hybridization

Formalin-fixed paraffin-embedded tissue samples were handled according to the manufacturer’s protocol using RNAscope 2.0 HD Assay (Advanced Cell Diagnostics [ACD], Hayward, CA, cat. no. 310033). In brief, FFPE sections of VAT, SCAT, normal myometrium (M-NP), myometrium from pregnant women (M-P), and testis (as a positive control for FSHR) tissue sections were deparaffinized in xylene (2 × 5 min), 100% EtOH (2 × 1 min) and air-dried for 5 min at room temperature. Each section was treated with hydrogen peroxide for 10 min at RT, then washed twice in distilled water. Slides were boiled in antigen retrieval buffer for 15 min and submerged in distilled water immediately thereafter. Next, the slides were washed in 100% EtOH and air-dried. For each section, barriers were drawn with the hydrophobic pen, and protease was applied for 30 min at 40 ºC in HybEZ™ Oven (ACD). The slides were washed twice in distilled water. Probes for the targeted transcripts (human FSHR: ACD-408101) were applied as well as probes for positive (cyclophilin B–PPIB, a housekeeping gene, human ACD-313901), positive control probe for low abundance transcripts (HS-POLR2A, ACD-310451) and negative controls (DapB – negative control probe targeting bacteria gene, ACD-310043). Then, slides were incubated at 40 ºC for 2 h in the oven and washed 2 × 2 min in the wash buffer. Thereafter, hybridization amplifiers (AMPs) were applied for 30 min (AMP 1, 3, 5) or 15 min (AMP 2, 4, 6) at 40 ºC (AMP 1–4) or room temperature (AMP 5 and 6) with double washing in between every step. After the last washing, equal volumes of BROWN-A and BROWN-B reagents were combined and applied to the sections for 10 min at RT. After double washing with distilled water, slides were counterstained in 50% Gill’s hematoxylin (Vector Laboratories, Burlingame, CA, USA) for 2 min, then washed in 0.02% ammonia water for 10 s and twice in distilled water. Dehydrated slides (2 × 2 min in 70% EtOH, 2 × 2 min in 100% EtOH, and 5 min in xylene) were mounted with Pertex (Histolab Products, Göteborg, Sweden). The RNAScope slides were digitally scanned by a P1000 Pannoramic scanner (3DHistech, Budapest, Hungary).

### Western blot

Adipocytes were seeded onto a 6-well plate (90000 cells/well) and labor myometrium explants were plated onto 24-well plates, incubated overnight in culture medium, and starved in stimulation medium (phenol-free DMEM/F12 with 0.5% dFBS and P/S solution) for 4 h. Cells and explants were stimulated without (control) or with 1, 10, 100, 1000 IU/L rhFSH for 15, 30 and 60 min and collected on RIPA buffer (Sigma Aldrich, cat. no. R0278) with the addition of Protease Inhibitor Cocktail (Sigma Aldrich, cat. no. P8340). Explants were additionally homogenized using ULTRA-TURRAX^®^. Total protein concentration was measured with a BCA Protein Assay Kit (Sigma Aldrich, cat. no. 71285-M). To analyze FSH-induced AKT pathway activation of adipocytes and myometrium, equal amounts of total proteins (25 µg) were separated on 10% polyacrylamide gels (1.5 h, 130 V, 4 °C). Proteins were transferred into nitrocellulose membranes (semi-dry transfer, 30 min, 15 V). Membranes were blocked in 5% non-fat dry milk in TBS with 0.05% Tween (TBST) solution for 1 h in RT and incubated overnight (4 °C with gentle agitation) with primary antibodies: anti-total AKT (Cell Signaling Technology, CST, Danvers, MA, USA, cat. no. 9272), anti-phospho-AKT (Ser473, CST, cat. no. 9271) and anti-GAPDH (housekeeping, Abcam, Cambridge, UK, cat. no. ab8245). Membranes were washed with TBST 3 × 5 min and incubated with anti-rabbit HRP-linked (CST, cat. no. 7074, for anti-AKT and anti-pAKT antibodies) or anti-mouse HRP-linked (CST, cat. no. 7076, for anti-GAPDH antibody) secondary antibody for 1 h at RT with gentle agitation. Membranes were washed 3 × 5 min with TBST and the Amersham Biosciences ECL detection system (GE Healthcare, Chicago, IL, USA, cat. no. 28980926) was used for protein visualization. Pictures were taken using the ChemiDoc MP Imaging System (Bio-Rad).

### Statistical analysis

Results were expressed as mean ± SEM. All data series were checked for consistency with Gaussian distribution by the D’Agostino-Pearson normality test. Statistical significance was assessed by one-way ANOVA with the post hoc Bonferroni test for cAMP analysis or by Tukey’s post-hoc or Mann-Whitney U test for contraction studies using GraphPad PRISM v. 9.0 (GraphPad Software, Inc). *P* ≤ 0.05 was considered as statistically significant.

## Results

### FSHRs are not expressed in human myometrium, adipose tissues, and adipocytes

**Fig. 1:**
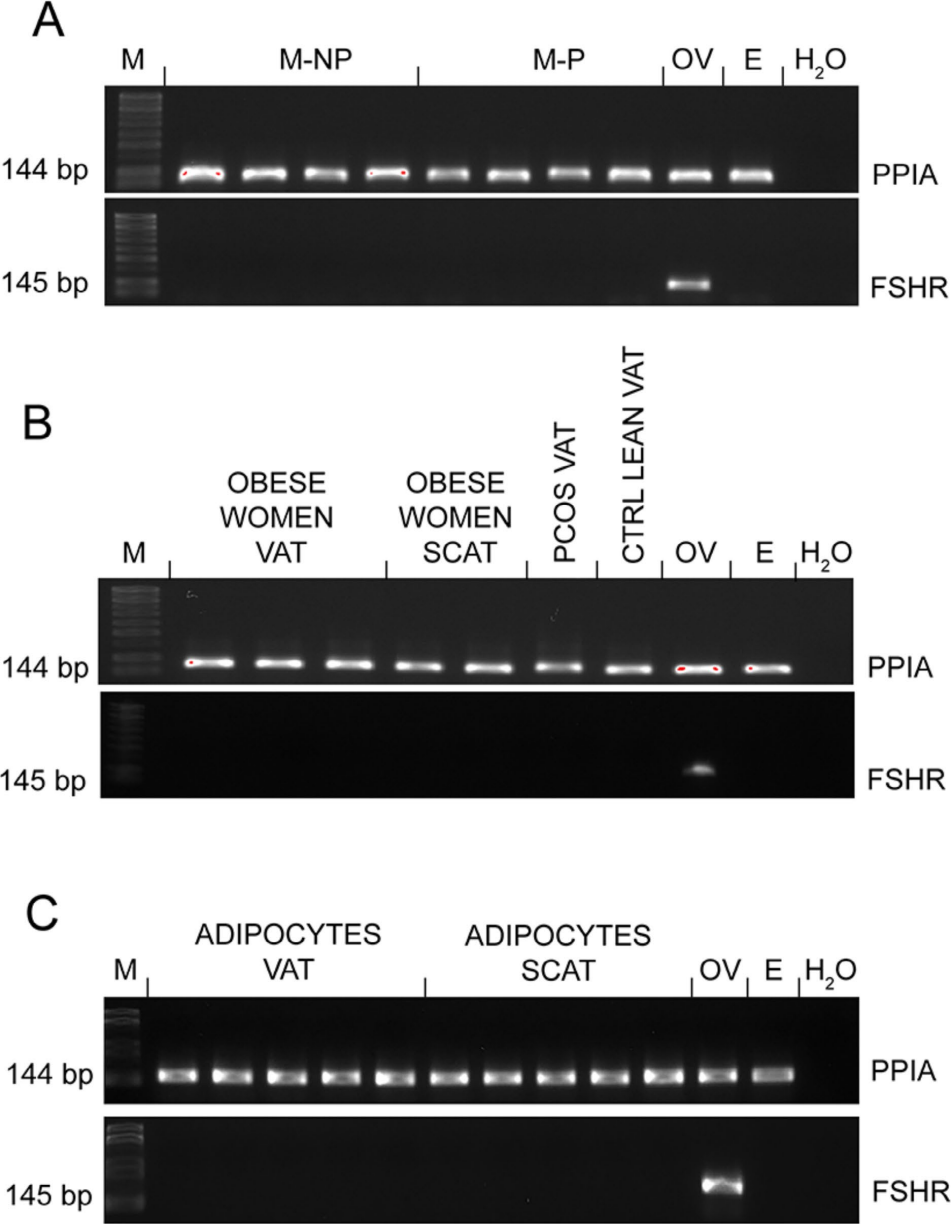
*FSHR* expression profile in the human myometrium, adipose tissue, and adipocytes. The qPCR analysis in 2% agarose gel of housekeeping gene *PPIA* and *FSHR* in (**A**) non-pregnant and pregnant myometrium, ovary and proliferative endometrium; (**B**) visceral and subcutaneous adipose tissue from obese women, visceral adipose tissue from women with PCOS and healthy women; (**C**) adipocytes. Amplicon sizes are presented on the left. E, proliferative endometrium; FSHR, follicle-stimulating hormone receptor; H_2_O, nuclease-free water; M, marker; M-NP, not-pregnant myometrium; M-P pregnant myometrium; OV, ovary; PPIA, Peptidylprolyl isomerase A; SCAT, subcutaneous adipose tissue; VAT, visceral adipose tissue

To characterize the FSHR expression profile in human myometrium, we analyzed myometrial tissue samples from non-pregnant (M-NP) and pregnant (M-P) women. *FSHR* expression was not detected in either M-NP or M-P tissues (Fig. 1A). Next, we checked the *FSHR* expression levels in visceral (VAT) and subcutaneous (SCAT) adipose tissue samples from obese and lean women, VAT from women with polycystic ovary syndrome (PCOS), and adipocytes isolated from VAT and SCAT. At first, we confirmed that isolated pre-adipocytes successfully differentiated into mature adipocytes, by checking this mature adipocyte markers peroxisome proliferator-activated receptor gamma (PPARG), CCAAT enhancer binding protein alpha (CEBP/A), CCAAT enhancer binding protein beta (CEBP/B), adiponectin (ADIPOQ), leptin (LEP), lipoprotein lipase (LPL) and perilipin 1 (PLIN1) (Supplemental Fig. 1). qPCR analyses did not show *FSHR* expression in VAT and SCAT from obese, VAT lean, and VAT PCOS women tissues (Fig. 1B), or in VAT and SCAT adipocytes (Fig. 1C). The human ovary (OV) used as a positive control showed clear *FSHR* expression, whereas proliferative endometrium (E) was *FSHR* negative (Fig. 1A-C).

**Fig. 2:**
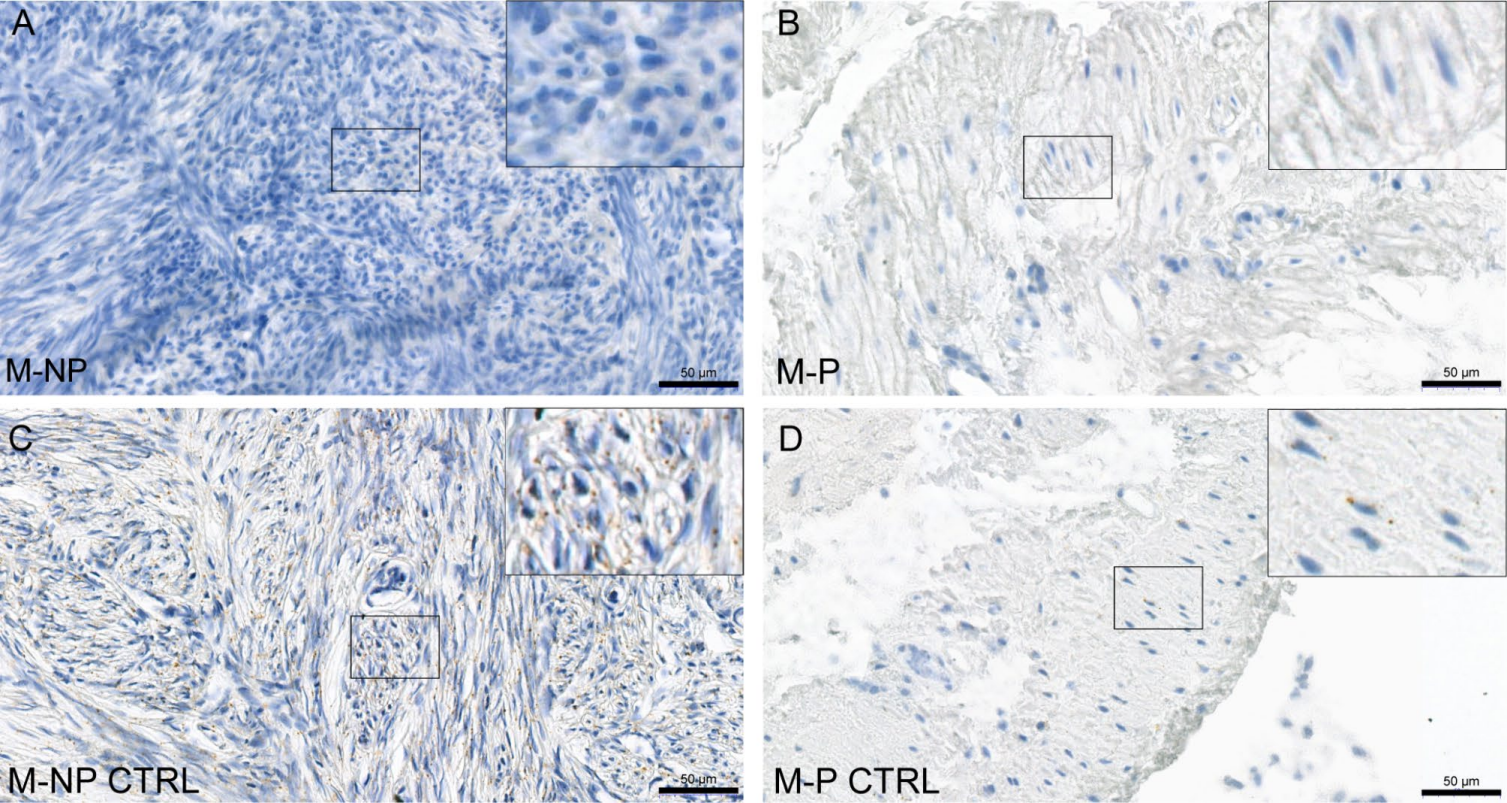
RNAscope in situ hybridization of *FSHR* mRNA transcripts in human myometrium and analysis of tissue sections quality and the specificity of RNAscope^®^ in situ hybridization. Localization of *FSHR* mRNA transcripts in human non-pregnant myometrium (**A**) and pregnant myometrium (**B**). Paraffin sections of human non-pregnant (**C**) and pregnant myometrium (**D**) were hybridized with a positive control probe complementary to human *Cyclophilin B*. Sections were counterstained with hematoxylin. M-NP, not-pregnant myometrium; M-NP CTRL, not-pregnant myometrium positive control; M-P pregnant myometrium; M-P CTRL, pregnant myometrium positive control

**Fig. 3:**
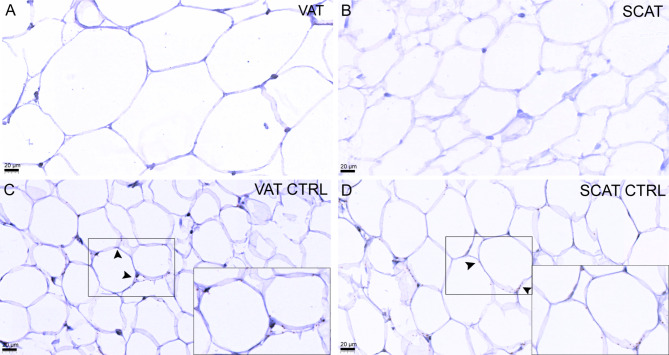
RNAscope in situ hybridization of *FSHR* mRNA transcripts in human adipose tissue and analysis of tissue sections quality and the specificity of RNAscope^®^ in situ hybridization. Localization of *FSHR* mRNA transcripts in visceral (**A**) and subcutaneous (**B**) adipose tissues. Paraffin sections of visceral (**C**) and subcutaneous (**D**) adipose tissues were hybridized with a positive control probe complementary to human *Cyclophilin B*. Sections were counterstained with hematoxylin. SCAT, subcutaneous adipose tissue; SCAT CTRL, subcutaneous adipose tissue positive control; VAT, visceral adipose tissue; VAT CTRL, visceral adipose tissue positive control

Next, we confirmed the absence of *FSHR* transcripts by RNAscope in situ hybridization using a specific FSHR probe. RNAscope analysis showed no *FSHR* transcripts in M-NP and M-P tissues (Fig. 2A-B). The *PPIB* probe used as a positive control for myometrium showed a clear positive signal in both M-NP and M-P tissues (Fig. 2C-D). Similarly, we found no *FSHR* transcript signals in the VAT and SCAT tissue sections (Fig. 3A-B). The quality of VAT and SCAT tissue sections and specificity of the RNA in situ hybridization assay were confirmed by the presence of *POLR2A* transcripts tested as a positive control probe (Fig. 3C-D). *DapB* was used as a negative control to test the quality of tissue sections and specificity of the RNAscope in situ hybridization assay, and it was not detected in any of the analyzed tissues (Supplemental Fig. 2A-D). FSHR transcripts were detected in Sertoli cells of the human testis used as a positive control (Supplemental Fig. 3A). *DapB* was used as a negative control, and it was not detected in testis (Supplemental Fig. 3B). We used as a positive control *Cyclophilin B*, which was expressed abundantly in the human testis (Supplemental Fig. 3C).

**Fig. 4:**
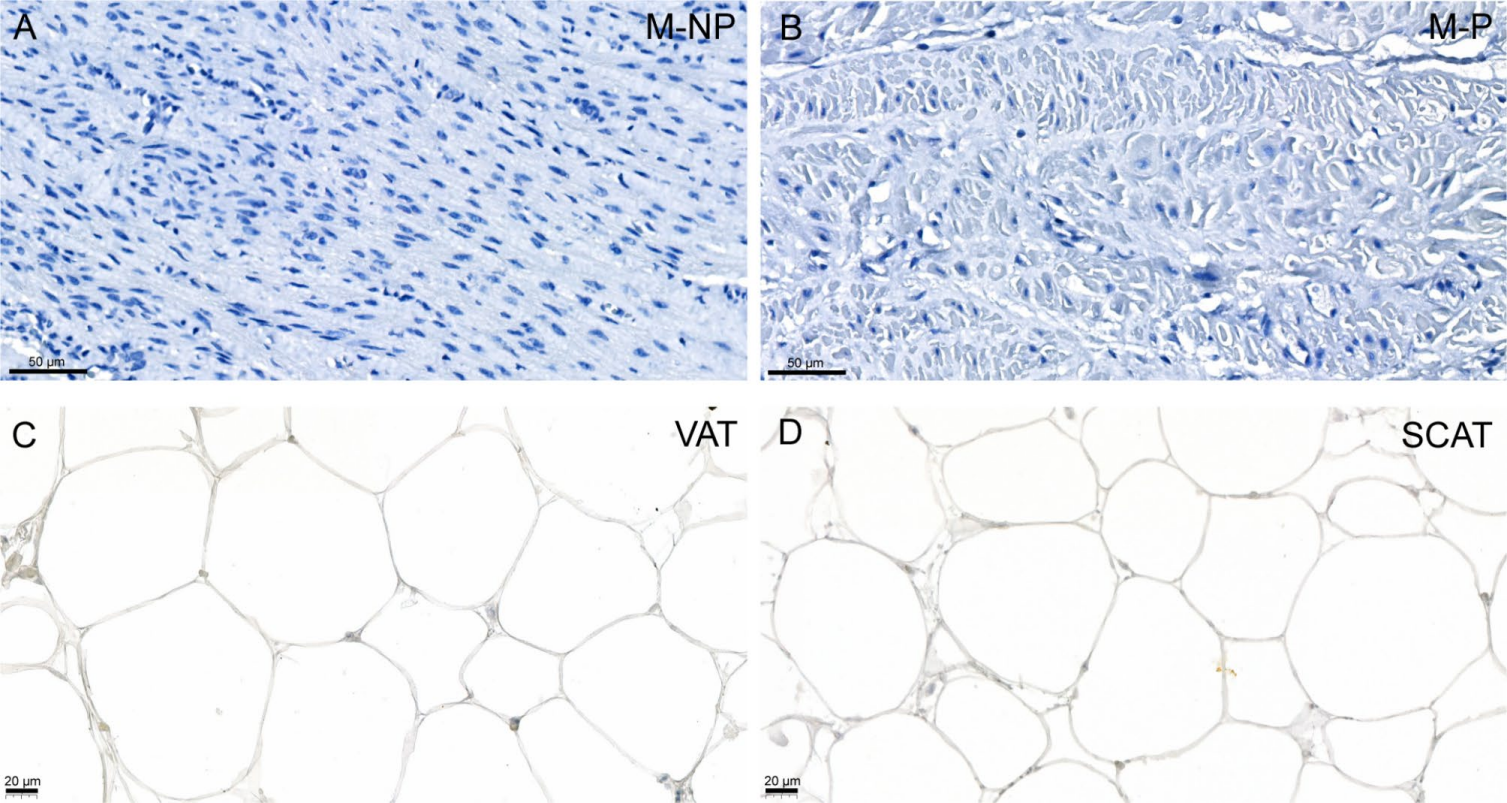
Immunohistochemical localization of FSHR in human myometrium and adipose tissue. Localization of FSHR protein in human non-pregnant myometrium (**A**), pregnant myometrium (**B**), visceral adipose tissue (**C**), and subcutaneous adipose tissue (**D**). Localization of FSHR protein in human testis (positive control) is shown in Supplemental Fig. 5A. M-NP, not-pregnant myometrium; M-P pregnant myometrium; SCAT, subcutaneous adipose tissue; VAT, visceral adipose tissue

Finally, we confirmed the lack of FSHR expression at the protein level using a mouse monoclonal FSHR323 antibody (Radu et al. 2010). We could not detect FSHR signals in M-NP and M-P tissues (Fig. 4A-B), neither in VAT nor SCAT (Fig. 4C-D). No FSHR staining was detected in the control IHC analysis without primary antibody (Supplemental Fig. 4A-D). Human testis used as positive control showed specific FSHR membrane staining in Sertoli cells (Supplemental Fig. 5A), whereas no staining was detected in the analysis without the primary antibody (Supplemental Fig. 5B).

### FSH does not affect the functional pathways in myometrium and adipose tissue

**Fig. 5:**
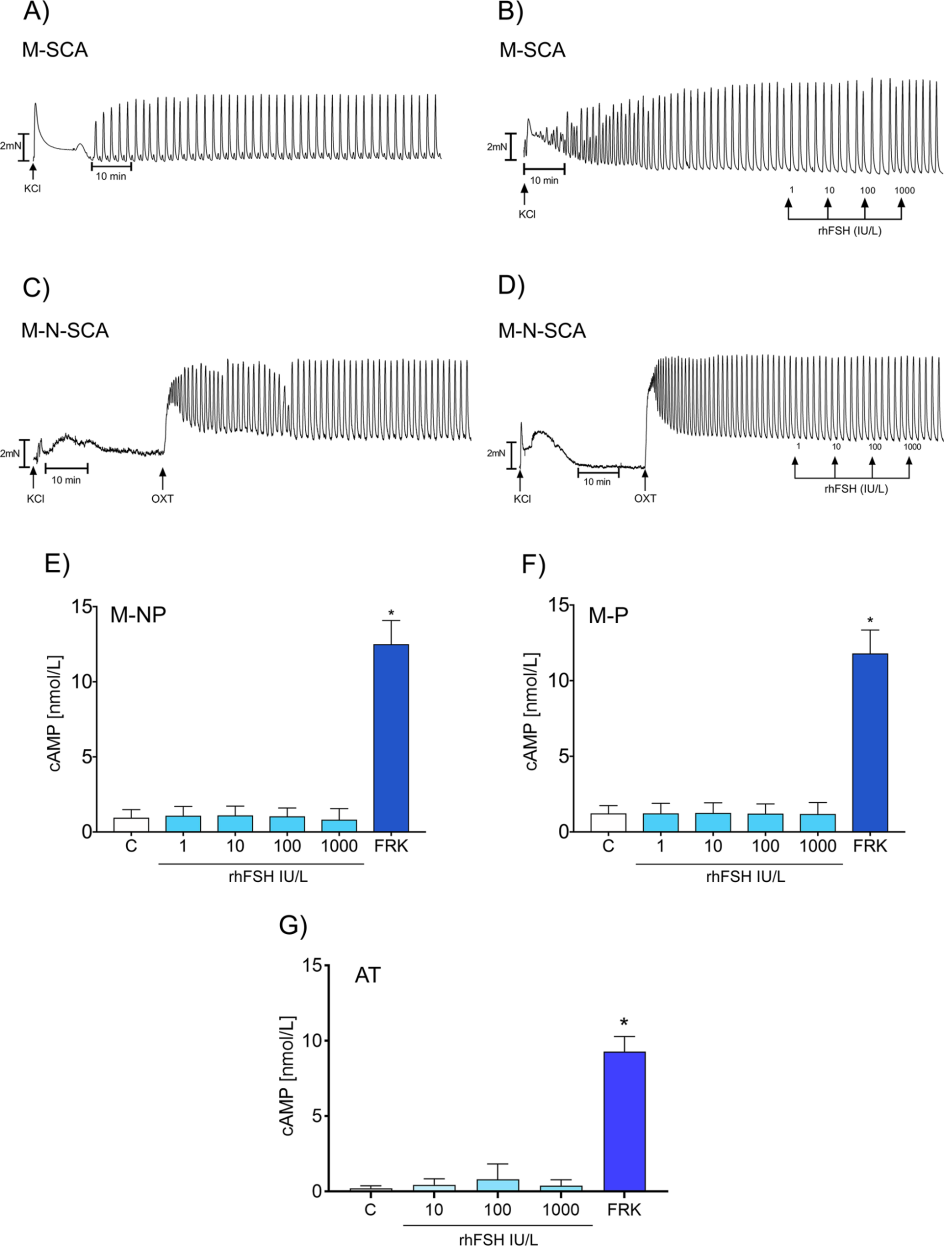
FSH effects on the contractile activity of pregnant myometrium and cAMP production by myometrium and adipose tissue. Records of the contractile activity of myometrium with a spontaneous contractile activity without rhFSH (**A**) and after rhFSH administration (**B**). Records of the contractile activity of myometrium without a spontaneous contractile activity without rhFSH (**C**) and after rhFSH administration (**D**). Contractile activity of myometrium was induced by the administration of oxytocin (**C**, **D**). Production of cAMP by non-pregnant myometrium (**E**), pregnant myometrium (**F**), and adipose tissue (**G**) after rhFSH stimulation. 10 µM FRK was used as a positive control. Each bar represents the mean ± SEM of three independent experiments with *n* = 6 per treatment. Asterisks indicate differences between control and stimulated cells (*P < 0.001). AT, adipose tissue; cAMP, cyclic adenosine monophosphate; FRK, forskolin; KCl, potassium chloride; M-NP, not-pregnant myometrium; M-N-SCA, myometrium without spontaneous contractile activity; M-P pregnant myometrium; M-SCA, myometrium with spontaneous contractile activity; OXT, oxytocin; rhFSH, recombinant human follicle-stimulating hormone

Despite the negative results of FSHR expression at mRNA and protein levels, we further strengthened our findings through functional experiments. We investigated the effect of rhFSH stimulation on the contractile activity of two groups of human myometrium, with spontaneous contractile activity (M-SCA) and without spontaneous contractile activity (M-N-SCA). In both groups studied, rhFSH did not change the contractile activity of myometrium (Fig. 5A-D). The addition of rhFSH showed no effect on myometrial contractions without spontaneous activity after oxytocin administration (Fig. 5C-D). AUC values did not change after rhFSH-stimulation of M-SCA and M-N-SCA (Supplemental Tables 2,3; Supplemental Fig. 6).

Furthermore, we studied the effect of rhFSH stimulation on cAMP production by M-NP and M-P as well as adipocytes. In all examined tissues, a rhFSH dose-response treatment showed no effects on cAMP production (Fig. 5E-G). Forskolin, used as a positive control, significantly increased cAMP production by M-NP and M-P tissues as well as by adipocytes (Fig. 5E-G).

**Fig. 6:**
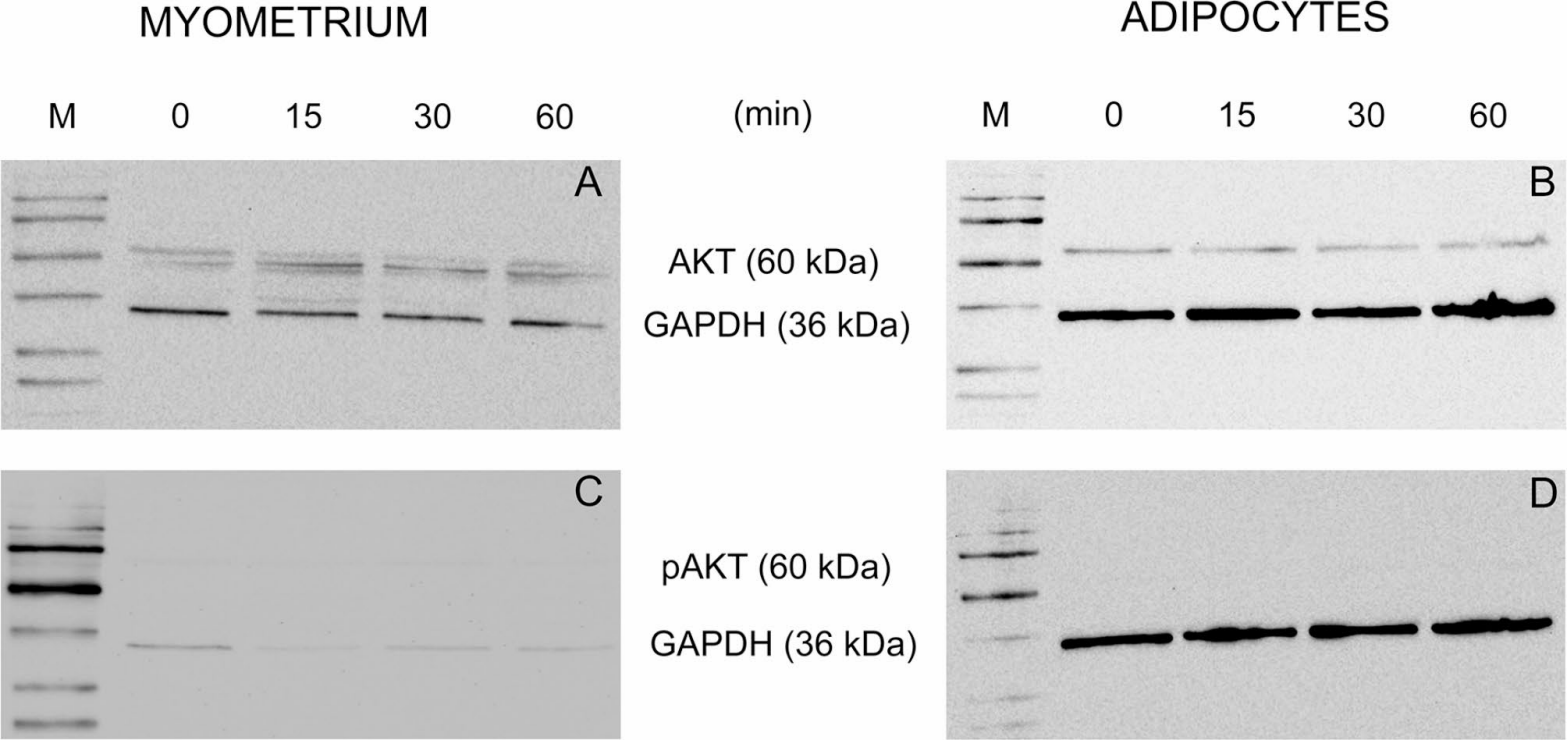
Western blot analysis of AKT phosphorylation in human myometrium extracts and adipocytes after rhFSH treatment. AKT protein expression in myometrium (**A**) and adipocytes (**B**). Phosphorylated AKT expression in myometrium (**C**) and adipocytes (**D**). Myometrium explants and adipocytes were stimulated with 1000 IU/L rhFSH for 15, 30, and 60 min. GAPDH was used as a loading control. AKT, protein kinase B; GAPDH, Glyceraldehyde 3-phosphate dehydrogenase; M, marker; pAKT, phosphorylated protein kinase B; rhFSH, recombinant human FSH

We also analyzed the activation of the AKT pathway in myometrium and adipocytes using Western blot analysis. There was no change in the total expression level of AKT (Fig. 6A-B) or phosphorylated AKT (pAKT) following rhFSH stimulation in the myometrium and/or adipocytes (Fig. 6C-D).

## Discussion

In principle, functional extragonadal FSHR expression could potentially serve as an excellent therapeutic target. However, most tissues shown to present with FSHR protein immunoreactivity have failed so far, upon mode scrutiny, to show amplification of FSHR transcripts (Ponikwicka-Tyszko et al. 2016; Stelmaszewska et al. 2016; Chrusciel et al. 2019). In the present study, we were unable to reproduce earlier results on FSHR expression in the myometrium (Stilley et al. 2014a, 2016) and adipose tissues (Liu et al. 2015, 2017; Cui et al. 2012, 2018). We did not find FSHR expression at the mRNA level in M-NP or M-P tissues. In contrast to our study, Stilley et al. (Stilley et al. 2014a) have shown that myometrium from term pregnancy expresses full-length *FSHR* mRNA, but unlike our study, the PCR products were not sequenced. Another study from the same group showed upregulation of the *FSHR* mRNA in pregnant-term human myometrium compared to nonpregnant myometrium, but in this study, the group size was small (*n* = 4) (Stilley et al. 2016). To confirm the absence of *FSHR* in M-NP and M-P in the present study, we used RNAscope in situ hybridization detection of FSHR transcripts, but the finding was negative in both groups. Moreover, using the monoclonal FSHR323 antibody (Radu et al. 2010), we could not localize FSHR expression at the protein level in M-NP and M-P tissues. In previous studies, the authors have used the same FSHR323 antibody to detect FSHR protein in M-NP and M-P (Stilley et al. 2014a, 2016). In normal M-NP, a positive FSHR signal has been detected in the endothelial cells of myometrium vessels and arterial smooth muscle, but only a very weak or traceable signal in myometrial muscle fibers (Stilley et al. 2014a). However, these studies (Stilley et al. 2014a, 2016) lacked the positive and negative tissue controls to confirm the specificity of the FSHR323 antibody (Chrusciel et al. 2019). We used the human testis as the positive control, which showed specific membrane FSHR staining in Sertoli cells. In the analysis of negative control without the primary antibody, we did not detect any FSHR staining. Moreover, we have previously shown no FSHR expression at mRNA and protein levels in normal human M-NP (Ponikwicka-Tyszko et al. 2016).

To further investigate the functionality of FSH/FSHR, we examined the effect of rhFSH stimulation on the contractile activity of human myometrium with and without spontaneous contractile activity. We showed that in neither M-SCA nor M-N-SCA groups, rhFSH stimulation changed the contractile activity of the myometrium. In contrast to our study, a former study hypothesized that FSH regulates myometrial contractility depending on the amount of FSHR in the myometrium (Stilley et al. 2016). In that study, in human and mouse M-NP, both expressing low levels of FSHR, stimulation with rhFSH resulted in quieting of contractile activity (Stilley et al. 2016). In contrast, in pregnant term nonlaboring myometrium, expressing higher levels of FSHR, FSH stimulated myometrial contractility (Stilley et al. 2016). However, these effects were observed only at supraphysiological rhFSH doses of 100–1000 ng/mL (136.43-1364.25 IU/L) (Stilley et al. 2016). In our study, at similar concentrations of up to 1000 IU/L of rhFSH, we could not confirm the earlier suppressive or stimulatory effects. FSH has also previously been shown to stimulate cAMP production in immortalized cells of human uterine myocytes derived from premenopausal nonpregnant myometrium (hTERT-HM) (Stilley et al. 2016). However, we found no rhFSH effect on cAMP production of normal or pregnant myometrium tissues. To support these negative findings, we also showed that rhFSH stimulation did not activate the AKT pathway. Our findings and the fact that women with inactivating FSHR mutation, lacking all potential FSHR expression, can maintain normal pregnancy until the term following ovum donation (Hovatta et al. 2002), seriously question the functional importance of extragonadal FSH action in myometrium.

We also examined the functional expression of FSHR in adipose tissue. However, we could not localize the FSHR expression in VAT, SCAT, and adipocytes at mRNA and protein levels. The negative FSHR in situ hybridization results were additionally strengthened by the qPCR results. In contrast to our study, expression of the FSHR has been shown in the human (Liu et al. 2015), mouse (Liu et al. 2015, 2017), and female chicken (Cui et al. 2012) adipose tissues and the 3T3-L1 mouse preadipocyte cell line (Liu et al. 2015; Cui et al. 2018). The abdominal adipose tissue of chicken expressed *Fshr* transcripts in the thin shell of the cytoplasm peripheral to the lipid locule (Cui et al. 2012). However, in these studies, the above-mentioned polyclonal anti-FSHR antibodies were used without proper positive and negative controls for immunohistochemical detection (Liu et al. 2015, 2017; Cui et al. 2018). We used the monoclonal antibody FSHR323 with convincing positive and negative controls. Besides lacking the appropriate positive and negative tissue controls, some studies have not sequenced their PCR products (Liu et al. 2017; Cui et al. 2018).

Recent studies have also shown that FSH stimulates lipid biosynthesis in vitro and in vivo by upregulating the expression of FSHR (Cui et al. 2012). In this study, the authors hypothesized that FSH regulates lipid accumulation in adipocytes by increasing gene expression related to fatty acid and retinol metabolism and PPAR signaling pathways (Cui et al. 2012). In vitro studies on the 3T3-L1 cell line and primary human adipocytes showed that FSH may directly stimulate lipid droplet formation but did not affect adipocyte hyperplasia (Liu et al. 2015). In contrast to the ovary, FSH could act through the Gαi/Ca^2+^/CREB pathway in human and 3T3-L1 cell line preadipocytes, suppress cAMP accumulation, and promote lipid biosynthesis (Liu et al. 2015). Furthermore, studies in gonadectomized mice have shown a significant increase in body weight and fat mass, which has been inhibited by treatment with a gonadotropin-releasing hormone (GnRH) agonist (GnRHa) but not FSH + GnRHa (Liu et al. 2015). Another study has shown that blockage of the FSH signaling with a specific polyclonal FSH antibody may also reduce obesity in ovariectomized mice (Liu et al. 2017). However, our previous results contradict these results, since we observed no reduction of adipose tissue in transgenic mice expressing Simian Virus 40 T antigen under inhibin-α promoter treated with a GnRH antagonist in conjunction with gonadal or adrenocortical tumor treatment (Vuorenoja et al. 2009; Doroszko et al. 2019). The lack of rhFSH-stimulated cAMP production and AKT phosphorylation further supports our negative results regarding the functional FSHR expression in adipocytes. Moreover, a clinical study conducted on men showed that FSH suppression in combination with gonadal steroids does not reduce body fat (Finkelstein et al. 2013). In light of these results, it therefore appears that the functional expression of FSHR in adipose tissue remains a matter of serious controversy.

The issue of extragonadal FSHR expression and its functionality remains contentious within the scientific community. Contradictory findings necessitate further independent and critical analyses and problems with the reproducibility of results indicate a great need for a more critical analysis of extragonadal FSHR expression data (Chrusciel et al. 2019). Results without additional sensitive methodological confirmation, often based only on IHC data with insufficiently validated antibodies may lead to potentially erroneous conclusions. In several studies, the high and abundant FSHR expression at the protein level was not accompanied by clear amplification of FSHR mRNA (Stilley et al., 2014b; Stelmaszewska et al. 2016). Moreover, when qPCR products were not sequenced, the rhFSH doses used in functional studies were supraphysiological, and the studies lacked adequate positive and negative controls, correct interpretation of data may become very difficult. To enhance the reliability and validity of FSHR IHC/IF and functional studies, the use of other methods, such as RNAScope in situ hybridization, along with a validated set of positive and negative probes is recommended (Chrusciel et al. 2019). It would be important to restrict rhFSH doses to physiological concentrations in functional studies, as only this approach ensures that the observed effects are relevant to normal biological functions and reduces the risk of artefactual results. More rigorous study design and appropriate controls will allow us to eliminate further discrepancies in results. Therefore, it is important to analyze the data more critically, carefully verify them, and publish all results, even negative ones.

## Conclusions

Taken together, we could not confirm the FSHR expression at mRNA and protein levels in human M-NP and N-P, as well as in VAT, SCAT, and adipocytes. rhFSH stimulation showed no effect on cAMP production or phosphorylation of AKT in myometrium and adipose tissues, or contractile activity in M-NP and N-P. Consequently, our data do not support any of the data on FSHR expression and function in uterine tissue, its contractility, or its effects on adipocytes. Therefore, the concept of the FSHR signaling pathway regulating myometrial contractility in pregnancy remains controversial. Additionally, our results suggest that FSH does not affect the functional pathways in adipose tissue. A major caution is thus needed when interpreting the existing data on the functionality of extragonadal FSH-FSHR signaling. More independent studies, with a variety of methodological approaches, are necessary to validate the FSHR expression and function.

## Electronic supplementary material

Below is the link to the electronic supplementary material.

Supplementary Material 1

## Data Availability

No datasets were generated or analysed during the current study.

## Abbreviations

ADIPOQ: Adiponectin
AT: Adipose Tissue
cAMP: cyclic Adenosine Monophosphate
CEBP/A: CCAAT Enhancer Binding Protein Alpha
CEBP/B: CCAAT Enhancer Binding Protein Beta
CTRL: Control
E: proliferative Endometrium
FRK: Forskolin
FSH: Follicle-Stimulating Hormone
FSHR: Follicle-Stimulating Hormone Receptor
H_2_O: Nuclease-Free Water
KCl: potassium Chloride
LEP: Leptin
LPL: Lipoprotein Lipase
M: Marker
M-NP: Not-Pregnant Myometrium
M-N-SCA: Myometrium without Spontaneous Contractile Activity
M-P: Pregnant Myometrium
M-SCA: Myometrium with Spontaneous Contractile Activity
OV: Ovary
OXT: Oxytocin
PCOS: Polycystic Ovary Syndrome
PLIN1: Perilipin 1
PPARG: Peroxisome Proliferator-Activated Receptor Gamma
PPIA: Peptidylprolyl Isomerase A
P/S: Penicillin/Streptomycin
rhFSH: recombinant human Follicle-Stimulating Hormone
SCAT: Subcutaneous Adipose Tissue
VAT: Visceral Adipose Tissue
