# Supplementary material for: Revisiting the follicle-stimulation hormone receptor expression and function in human myometrium and adipose tissue
Source: Mol Med. 2024 Dec 4;30:241. doi: 10.1186/s10020-024-01015-2 (PMC11619181; doi:10.1186/s10020-024-01015-2)
Supplement: Supplementary file 1 — Supplementary Material 1 [file 10020_2024_1015_MOESM1_ESM.docx]

**Revisiting the follicle-stimulation hormone receptor expression and function in human myometrium and adipose tissue**

Ewelina Palak^1,2,a^_,_ Donata Ponikwicka-Tyszko^1,2,a^, Kamila Pulawska-Moon^2^, Maria Sztachelska^1^, Gabriela Milewska^3^, Beata Modzelewska^4^, Tomasz Kleszczewski^4^, Maria L. Koivukoski^2^, Piotr Bernaczyk^5^, Hady Razak Hady^6^, Piotr Gołaszewski^6^, Aleksandra N. Lupinska^1^, Marek Kulikowski^7^, Adam Lemancewicz^7^, Ilpo T. Huhtaniemi^8^, Slawomir Wolczynski^1,3^, Nafis A. Rahman^2,3⁎^

^1^Department of Biology and Pathology of Human Reproduction, Institute of Animal Reproduction and Food Research, Polish Academy of Sciences, 10-748 Olsztyn, Poland;

^2^Institute of Biomedicine, Research Centre for Integrative Physiology and Pharmacology University of Turku, 20520 Turku, Finland;

^3^Department of Reproduction and Gynecological Endocrinology, Medical University of Bialystok, 15-276 Bialystok, Poland;

^4^Department of Biophysics, Medical University of Bialystok, 15-269 Bialystok, Poland;

^5^Department of Medical Pathomorphology, Medical University of Bialystok, 15-269 Bialystok, Poland;

^6^1^st^ Clinical Department of General and Endocrine Surgery, Medical University of Bialystok, 15-269 Bialystok, Poland;

^7^ Department of Perinatology, Medical University of Bialystok, 15-269 Bialystok, Poland;

^8^Institute of Reproductive and Developmental Biology, Imperial College London, London W12 0NN,

^a^ equal contribution

* Correspondence: [nafis.rahman@utu.fi](mailto:nafis.rahman@utu.fi)


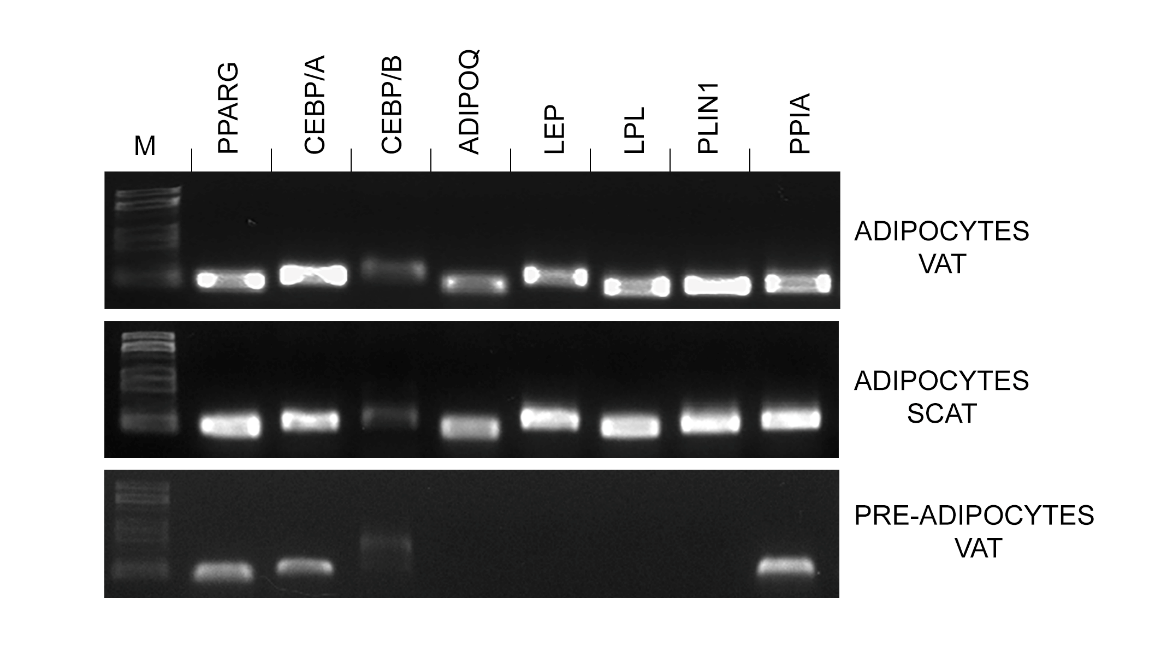

**Figure S1.** Expression profile of mature adipocyte markers in adipocytes (SCAT and VAT) and pre-adipocytes. ADIPOQ, adiponectin; CEBP/A, CCAAT enhancer binding protein alpha; CEBP/B, CCAAT enhancer binding protein beta; LEP, leptin; LPL, lipoprotein lipase; PLIN1, perilipin 1; PPARG, peroxisome proliferator activated receptor gamma; PPIA, peptidylprolyl isomerase A (housekeeping gene); SCAT, subcutaneous adipose tissue; VAT, visceral adipose tissue;


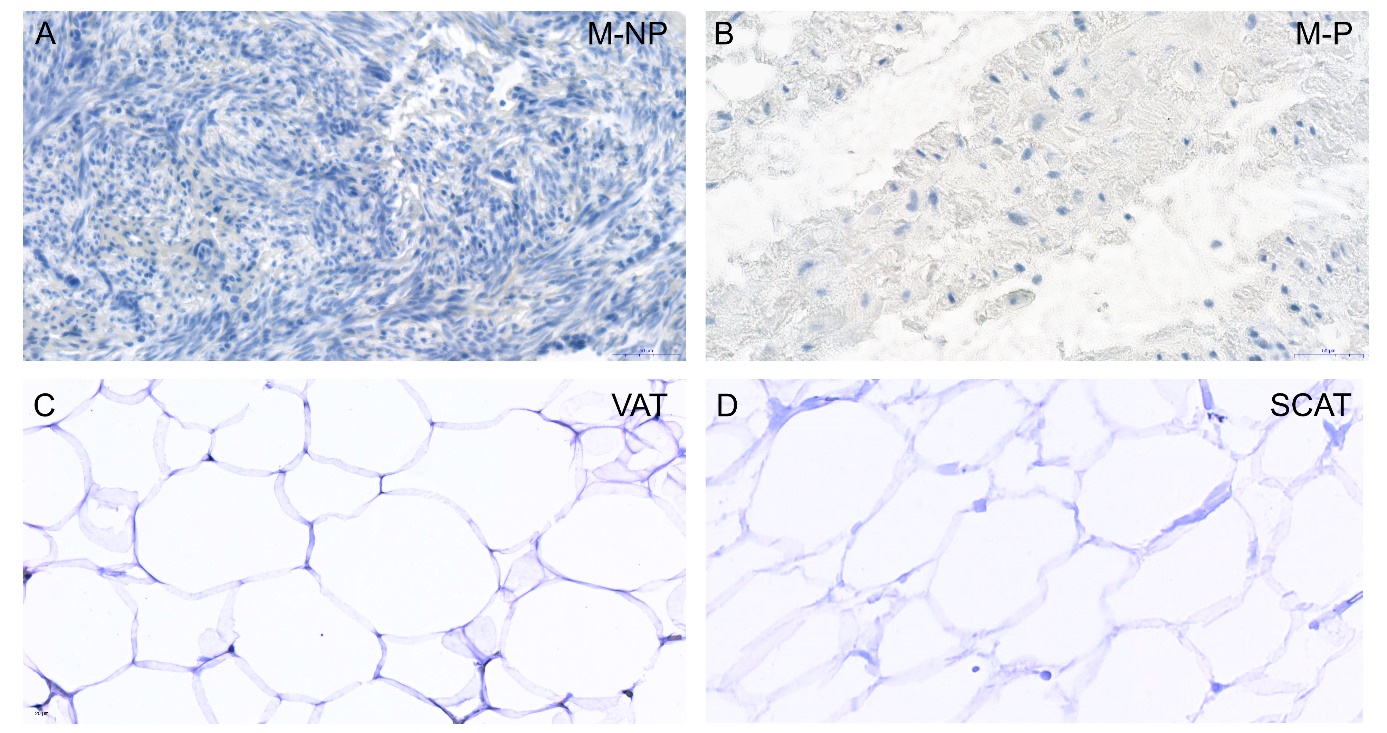
 **Figure S2.** Analysis of tissue section quality and the specificity of the control probes for RNAscope® *in situ* hybridization. Paraffin sections of human M-NP (A), M-P (B), VAT (C) and SCAT (D) were hybridized with a negative control probe that targets bacteria *DapB* gene. Sections were counterstained with hematoxylin. M-NP, not-pregnant myometrium; M-P pregnant myometrium; SCAT, subcutaneous adipose tissue; VAT, visceral adipose tissue;


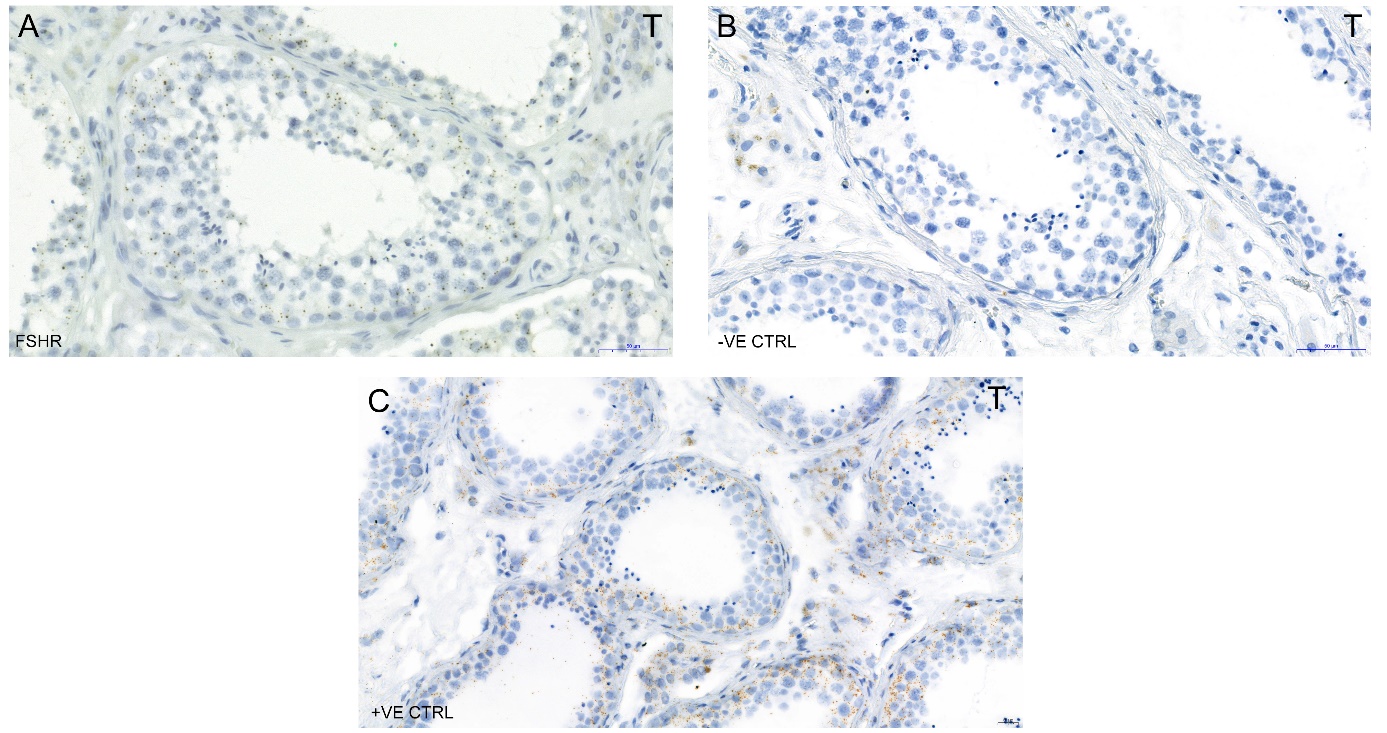


**Figure S3.** RNAscope in situ hybridization analysis for FSHR mRNA transcripts in human testis and analysis of tissue sections quality and the specificity of the negative and positive probes for RNAscope® *in situ* hybridization. Localization of FSHR mRNA transcripts in human testis (A). Paraffin sections of human testis were hybridized with a negative control probe that targets bacteria DapB gene (B) and a positive control probe complementary to human Cyclophilin B (C). Sections were counterstained with hematoxylin. +VE CTRL; positive control; -VE CTRL; negative control; FSHR, follicle-stimulating hormone receptor; T, testis


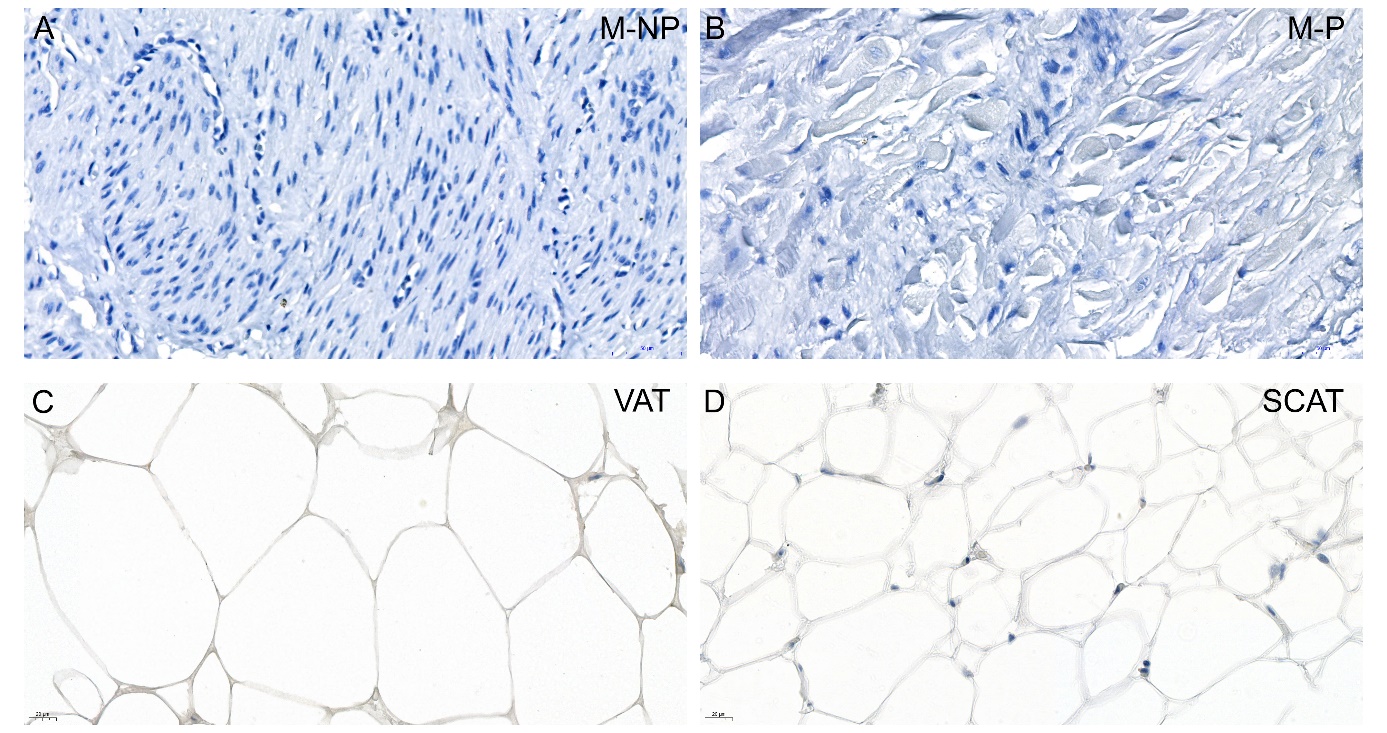

**Figure S4.** Analysis of the primary antibody specificity in immunohistochemical studies. Paraffin sections of human M-NP (A), M-P (B), VAT (C), and SCAT (D) were incubated with 3% BSA and DAKO EnVision+ System – HRP labeled polymer. Sections were counterstained with hematoxylin. M-NP, not-pregnant myometrium; M-P pregnant myometrium; SCAT, subcutaneous adipose tissue; VAT, visceral adipose tissue;


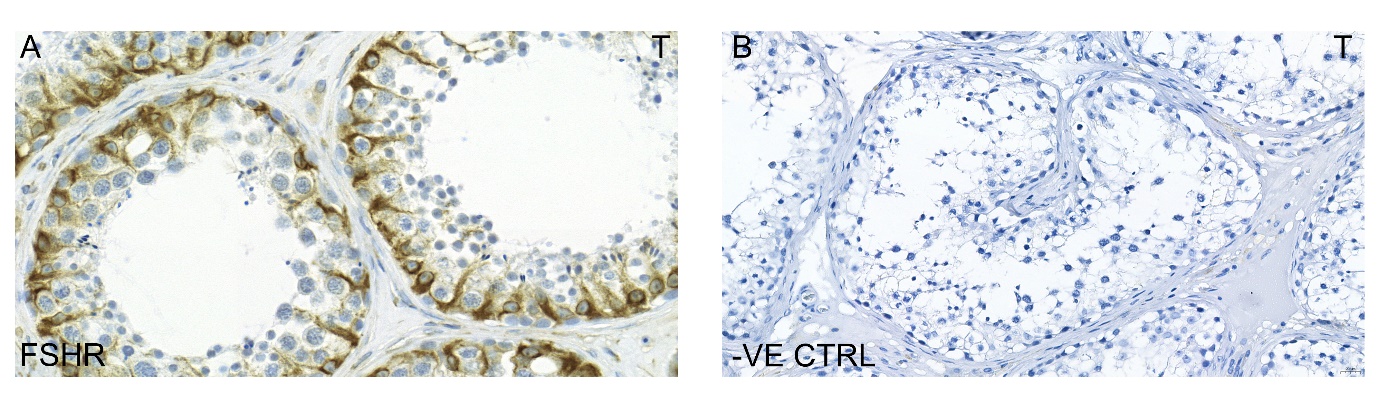


**Figure S5.** Immunohistochemical localization of FSHR in human testis and analysis of the primary antibody specificity. Localization of FSHR protein in human testis (A). Paraffin sections of the human testis (B) were incubated with 3% BSA and DAKO EnVision+ System – HRP labeled polymer. Sections were counterstained with hematoxylin. -VE CTRL; negative control; FSHR, follicle-stimulating hormone receptor; T, testis


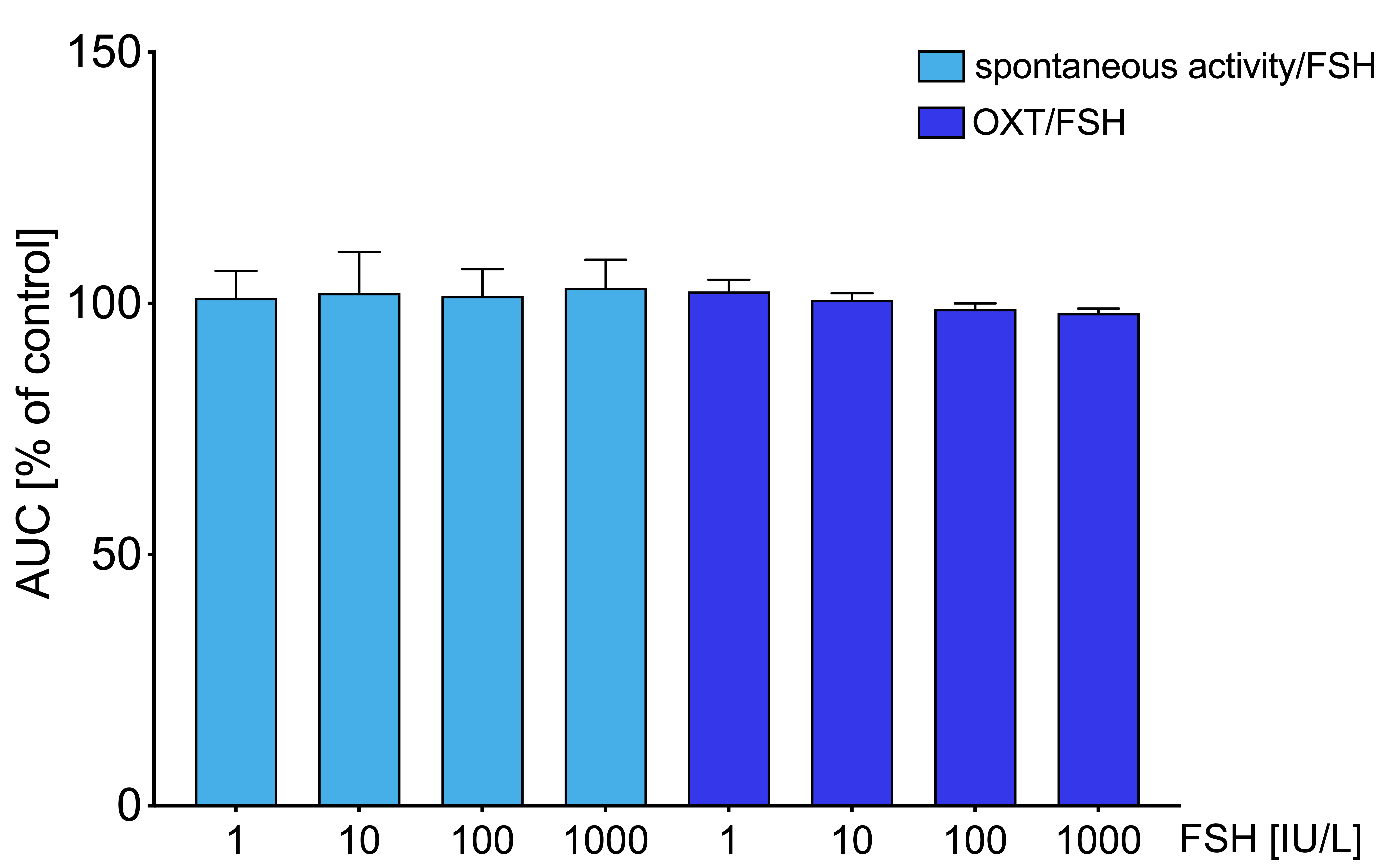


**Figure S6.** AUC values after FSH administration relative to controls. 100% - AUC for spontaneous contractile activity

**Table S1.** Primers sequences used for gene expression analysis.

| **Gene symbol** |  | **Primer Sequence (5’-3’)** | **Product size (bp)** | **NCBI**  **references** |
| --- | --- | --- | --- | --- |
| *PPIA** | F | GCCAAGACTGAGTGGTTGGATG | 144 | NM_021130.4 |
|  | R | GAGTTGTCCACAGTCAGCAATGG |  |  |
| *FSHR* | F | TGGGCTCAGGATGTCATCATCGGA | 145 | NM_000145.3 |
|  | R | TGGATGACTCGAAGCTTGGTGAGG |  |  |
| *ADIPOQ* | F | CTGACATCAGGGCTCAGGAT | 66 | NM_001177800.2 |
|  | R | ATGACCGGGCAGAGCTAATA |  |  |
| *CEBP/A* | F | AAAGGGGTGGAAACATAGGG | 95 | NM_001287435.1 |
|  | R | GGAGAGGCGTGGAACTAGAG |  |  |
| *CEBP/B* | F | CGGGCTCAGGAGAAACTTTA | 93 | NM_001285879.1 |
|  | R | TATTAGTGAGGGGGCTGGTG |  |  |
| *LEP* | F | CCCATCCAAAAAGTCCAAGA | 122 | NM_000230.3 |
|  | R | ATGAAGTCCAAACCGGTGA |  |  |
| *LPL* | F | CAGCCAGGATGTAACATTGG | 90 | NM_000237.3 |
|  | R | CTCGTGGGAGCACTTCACTA |  |  |
| *PLIN1* | F | GAGTGAGTGTTGGGGTCCTG | 104 | NM_002666.5 |
|  | R | CCTTTGTTGACTGCCATCCT |  |  |
| *PPARG* | F | CGGAACACGTGCAGCTACTG | 65 | NM_138712.5 |
|  | R | GAGCGGGTGAAGACTCATGTC |  |  |

**Table S2**. AUC values versus FSH concentration for tissues that showed spontaneous contractile activity (100% - AUC for spontaneous contractile activity)

| FSH concentration IU/L | 1 | 10 | 100 | 1000 |
| --- | --- | --- | --- | --- |
| AUC % in relation to the spontaneous activity (Mean± SEM) | 98,97±1,136 | 98,23±1,599 | 98,75±1,254 | 100,5±0,9493 |

For all concentrations, the differences in AUC versus control AUC were not statistically significant (p >0.005)

**Table S3.** AUC values versus FSH concentration for tissues that showed spontaneous contractile activity (100% - AUC for oxytocin-induced contractile activity)

| FSH concentration IU/L | 1 | 10 | 100 | 1000 |
| --- | --- | --- | --- | --- |
| AUC % in relation to the oxytocin induced contractile activity (Mean± SEM) | 98,85 ± 0,2992 | 98,13 ± 0,3356 | 96,71 ± 0,5097 | 95,71 ± 0,5112 |

For all concentrations, the differences in AUC versus control AUC were not statistically significant (p >0.005)
